# Patient-ventilator asynchronies: may the respiratory mechanics play a role?

**DOI:** 10.1186/cc12580

**Published:** 2013-03-25

**Authors:** Annalisa Carlucci, Lara Pisani, Piero Ceriana, Alberto Malovini, Stefano Nava

**Affiliations:** 1 Respiratory Intensive Care Unit, IRCCS Fondazione S. Maugeri, Via Maugeri 10, Pavia, 27100, Italy; 2 Respiratory and Critical Care Unit, S. Orsola-Malpighi Hospital, Via Pietro Albertoni 15, Bologna, 40138, Italy; 3 Laboratorio di Informatica e Sistemica per la Ricerca Clinica, IRCCS Fondazione S. Maugeri, Via Maugeri 10, Pavia, 27100, Italy; 4 Alma Mater University Department of Clinical, Integrated and Experimental Medicine (DIMAS), Respiratory and Critical Care Unit, S. Orsola-Malpighi Hospital, Via Pietro Albertoni 15, Bologna, 40138, Italy

## Abstract

**Introduction:**

The mechanisms leading to patient/ventilator asynchrony has never been systematically assessed. We studied the possible association between asynchrony and respiratory mechanics in patients ready to be enrolled for a home non-invasive ventilatory program. Secondarily, we looked for possible differences in the amount of asynchronies between obstructive and restrictive patients and a possible role of asynchrony in influencing the tolerance of non-invasive ventilation (NIV).

**Methods:**

The respiratory pattern and mechanics of 69 consecutive patients with chronic respiratory failure were recorded during spontaneous breathing. After that patients underwent non-invasive ventilation for 60 minutes with a "dedicated" NIV platform in a pressure support mode during the day. In the last 15 minutes of this period, asynchrony events were detected and classified as ineffective effort (IE), double triggering (DT) and auto-triggering (AT).

**Results:**

The overall number of asynchronies was not influenced by any variable of respiratory mechanics or by the underlying pathologies (that is, obstructive vs restrictive patients). There was a high prevalence of asynchrony events (58% of patients). IEs were the most frequent asynchronous events (45% of patients) and were associated with a higher level of pressure support. A high incidence of asynchrony events and IE were associated with a poor tolerance of NIV.

**Conclusions:**

Our study suggests that in non-invasively ventilated patients for a chronic respiratory failure, the incidence of patient-ventilator asynchronies was relatively high, but did not correlate with any parameters of respiratory mechanics or underlying disease.

## Introduction

The patient-ventilator interaction is an important clinical challenge during both invasive mechanical ventilation (IMV) and non-invasive ventilation (NIV). Asynchrony is present when there is a mismatch between the neural (patient) and mechanically (ventilator) assisted breaths and/or when ventilator flow delivery is inadequate to match the patient's ventilatory flow demand despite a matched inspiratory time [1]. Asynchrony is common in patients receiving NIV for acute respiratory failure, during which the presence of air-leaks and of a high inspiratory support seem to be among the major determinants of the mismatch [2]. A high incidence of asynchrony is associated with a prolonged duration of mechanical ventilation and a higher rate of tracheostomy during assisted IMV [3] and to a higher discomfort in patients receiving NIV for acute respiratory failure [2].

A European survey [4] performed in 2002 showed that about 22,000 patients are ventilated at home with either IMV or NIV. In patients with chronic respiratory failure secondary to chronic obstructive pulmonary disease (COPD) or neuromuscular disorders, the settings of pressure support (PS) and positive end-expiratory pressure (PEEP_e_), established on the basis of an invasive evaluation of lung mechanics and respiratory muscle function may result, when compared with a clinical adjustment, in a reduction of the patient's ineffective inspiratory efforts both during sleep and while awake [5,6]. These findings strongly suggest that respiratory mechanics and/or the type of underlying disease play a role in generation of asynchrony, but so far no study has systematically assessed whether or not patients' mechanical characteristics (that is, lung compliances, resistances and intrinsic positive end-expiratory pressures) may influence the presence of patient-ventilator mismatching. For the first time, we used the data of respiratory mechanics recorded during unsupported breathing with the primary aim to evaluate possible associations between the main asynchronies and respiratory mechanics, breathing pattern and respiratory effort in non-invasively ventilated patients with chronic respiratory failure, ready to be enrolled into a home ventilatory program. Secondary outcomes were to verify any possible association between asynchrony incidence and NIV tolerance as for the acute setting, and differences in the amount of asynchronies between obstructive and restrictive patients. We have chosen to assess respiratory mechanics during unsupported breathing since it has been speculated that several independent variables, like pulmonary compliance, resistances and intrinsic PEEP (strongly dependent on dynamic hyperinflation and therefore breathing pattern), may influence the behavior of the interaction between the patient and the ventilator [7]. Ventilator settings defined by the clinicians may deeply influence the breathing pattern and, therefore, do not allow us to discriminate *a priori *who will be the "problematic patient" to ventilate.

## Materials and methods

### Patients

This prospective, physiological study was performed on 69 consecutive patients undergoing pressure-support ventilation with non-invasive mechanical ventilation in our Respiratory Intensive Care Unit (ICU) and ready to be discharged with a home ventilatory program. The protocol was approved by the Central Ethics Committee Fondazione S. Maugeri of our Institution and all the patients gave written informed consent for their participation in the study. The patients had primarily been admitted to our unit: (i) to undergo a trial of NIV in the presence of an episode of acute respiratory failure or (ii) to undergo a domiciliary NIV treatment because of a chronic hypercapnic respiratory failure. In the former case, once clinical stability had been achieved, defined as no changes in arterial blood gases >10% in the last two days, and no need for new pharmacologic therapy and hemodynamic stability, the attending physician decided whether a particular patient was eligible for a home care program, based on our institute's decision tree. Briefly, the criteria used to determine the need for home NIV were: PaCO_2 _>50 mmHg, pH ≥ 7.35 breathing room air (after an exacerbation which required a NIV treatment), and/or polygraphic signs of nocturnal hypoventilation with daytime symptoms. Nocturnal hypoventilation was defined as the presence of tonic, profound desaturations occurring mainly during REM sleep and with more than 10% of total sleep time spent with SaO_2 _<90%. To eliminate a possible bias, NIV was delivered using a single type of ventilator, irrespective of the home-ventilator that the patients would have used once discharged. All patients were, in fact, ventilated with a "dedicated" NIV platform (BiPAP Vision, Philips Respironics Inc., Murrysville, PA, USA), in a pressure-support mode; settings were decided by the clinicians in charge of the patients and unaware of the aim of the study. The parameters are usually chosen to obtain a tidal volume between 6 and 8 ml/Kg, a reduction in respiratory rate vs spontaneous breathing, a good tolerance and a reduction of PaCO_2_. The back-up rate was set at the minimum value (that is, four breaths per minute), and the pressurization rate was set at 0.2 sec. Inspiration is flow triggered, and it is governed by an automatic algorithm which also controls the beginning of expiration and is named "autotrack". No further interventions were performed by the investigators. Exclusion criteria were sedation, sensorial impairment, diaphoresis and inability to swallow the gastro and esophageal balloons.

### Protocol

All patients were studied in a semi-recumbent position with a 45° bed head elevation. Baseline recordings of breathing pattern and respiratory mechanics were obtained during a 15-minute period of spontaneous quiet breathing. After that, a trial of NIV lasting 60 minutes was started by using the same oro-nasal mask to which patients were accustomed. The physiological recordings of patient-ventilator interaction were collected breath-by-breath during the last 15 minutes. A respiratory therapist took care to prevent air-leaks to avoid, as much as possible, the presence of this confounding factor in a study that was aimed mainly at studying the effect of respiratory mechanics. During the recording phase, patients breathed FiO_2 _sufficient to maintain the SpO_2 _value at an average of 93 to 94%.

### Measurements

Data on respiratory mechanics were obtained by passing through the nose two catheters with a distal balloon; one of them was positioned in the lower third of the esophagus and filled with 0.5 mL of air, the other was positioned in the stomach and was filled with 1 mL of air. Each catheter had a diameter of 2 mm, while the balloon was approximately 10 cm long and 1 cm wide. The proper position of the esophageal balloon was verified using the occlusion test [8]. Transdiaphragmatic pressure (P_di_) was calculated as the difference between gastric (P_ga_) and esophageal (P_es_) pressure. The pressure time integrals of the diaphragm were calculated per breath (PTP_di_), and per minute (PTP_di/min_). These latter are indices of oxygen consumption of the diaphragm [9]. The following additional variables were evaluated: (i) flow (V), measured by a heated pneumotachograph (Hans-Rudolph 3700, Kansas city, MO, USA) connected to the facial mask; (ii) tidal volume (V_T_) obtained by integration of the flow; (iii) inspiratory time (T_I_), expiratory time (T_E_), total respiratory time (T_TOT_) and spontaneous respiratory rate measured from the flow signal; and (iv) airway pressure (P_aw_), measured by a differential pressure transducer (± 300 H_2_O; Honeywell, Freeport, IL, USA) via a side port between the pneumotachograph and the facial mask.

For the baseline measurements, the pneumotachograph was placed immediately in front of a solid mouthpiece.

The dynamic intrinsic end-expiratory pressure (PEEP_dyn_) was obtained from the P_di _signal, as the delta value of P_di _from the raising of the signal until the beginning of flow [8]. Dynamic lung compliance (C_Ldyn_) and pulmonary resistance at mid-inspiratory volume (R_L_) were computed from transpulmonary pressure (P_L_), V and V_T _records as previously described [10].

### Patient-ventilator asynchrony

Asynchrony events, mainly ineffective efforts (IE), double triggering (DT) and auto-triggering (AT), were assessed by analysis of the flow, pressure and Pdi signals recorded over a 15-minute period during mechanical ventilation, as previously described [3]. Briefly, the three types of asynchrony were defined as follows:(i) IE were identified by a positive Pdi-tidal swing not followed by an assisted ventilatory cycle; (ii) DT was defined as two consecutive ventilator cycles separated by an expiratory time less than one-half the mean inspiratory time; (iii) AT was defined as a cycle delivered by the ventilator not associated with a contemporaneous Pdi swing.

The asynchrony index (AI) [3] was defined by the number of asynchrony events divided by the total respiratory rate computed as the sum of the number of ventilator cycles (patient-triggered) and of wasted efforts: asynchrony index (expressed in percentage) = number of asynchrony events/total respiratory rate (ventilator cycles + ineffective triggering) × 100. As previously reported [3], a high incidence of asynchrony was defined as an asynchrony index greater than 10%.

### NIV tolerance

The patient's tolerance to ventilation was evaluated using a modified visual analogue scale [11] at the end of the 60-minute NIV trial. This scale has four scores: 1 = very good, 2 = good, 3 = sufficient, 4 = bad. At the respiratory therapist's question: "How do you feel your breathing is at this moment?'', the patient answered by placing a finger on the number that best represented the intensity of his or her dyspnoea.

A possible association between the level of tolerance to NIV with the incidence of asynchronies was also analyzed.

### Statistical analysis

Data from the 15-minute period were averaged, and are expressed as medians and interquartile ranges for variables deviating from the normal distribution (*P*-value from the Shapiro test for normality <0.05); the mean ± standard deviation was reported for variables that did not deviate from the normal distribution (*P*-value from the Shapiro test for normality >0.05). Differences in terms of quantitative variables between binary subgroups were assessed by the non-parametric two-sided Wilcoxon rank-sum test, except for variables not deviating from the normal distribution for which the two-sided t-test was applied. The distribution of the asynchronies, according to the different underlying pathologies, was analyzed using Fisher's exact test. A formal *a priori *calculation of the sample size was not performed, since no data were available so far concerning the relationship between the occurrence of asynchronies and the respiratory mechanics. The number of patients to be enrolled has been, therefore, considered to be similar to the previous study [2], which reported a percentage of asynchronies in the NIV group (38%) high enough to detect any kind of correlation with respiratory mechanics. Because of the small number of patients affected by neuromuscular disease, for the statistical analysis these patients were considered in the "restrictive disease" group. *P*-values ≤ 0.05 were considered statistically significant. All statistical analyses were performed by the R statistical software [12].

## Results

Sixty-nine patients (47 males, mean age 65.3 ± 4) agreed to take part in the study. The cause for chronic respiratory failure was COPD for 30 patients (45%), restrictive thoraco-pulmonary disease (fibrothorax, kyphoscoliosis) for 31 patients (44%) and neuromuscular disease for 8 patients (11%). The mean values of arterial blood gases were the following: a PaO_2 _= 63 ± 12 (while breathing oxygen with a FiO_2 _= 33 ± 5%), PaCO_2 _= 62 ± 7, pH = 7.37 ± 0.01. The NIV was started 5 ± 3 days before the patients were enrolled in the study. There were no differences in inspiratory and expiratory pressure settings between patients divided according to the different pathologies (mean PS was 16.1 ± 3.3 in restrictive patients and 15.3 ± 3.7 in obstructive patients; median PEEP_e _was 4 (interquartile range = 2 to 4) in both groups). Data on respiratory pattern, inspiratory effort and respiratory mechanics according to the different pathologies are presented in Table 1 (lower part). Patients with obstructive disease had a higher PTPdi/min; that is an estimation of oxygen consumption of the diaphragm, vs those with restrictive disease, mainly due to a higher R_L _and PEEP_dyn_.

**Table 1:** Breathing pattern and respiratory mechanics according to the underlying respiratory disease

Variable	Restrictive diseases (*n *= 39)	Obstructive diseases (*n *= 30)	*P*
***Breathing pattern***			
Respiratory rate (breath/min)	25 (9)	19 (5)	**0.001**
V_T _(ml)	450 (379 to 625)	497 (403 to 631)	0.64
***Respiratory mechanics***			
C_Ldyn _(l/cmH_2_O)	0.05 (0.03 to 0.12)	0.06 (0.04 to 0.09)	0.38
R_L _(cmH_2_O/lxs)	5.7 (3.8 to 8.6)	15.8 (10.1 to 19.8)	**<0.001**
PEEP_dyn _(cmH_2_O)	0.8 (0.5 to 1.1)	1.8 (1.2 to 3)	**<0.001**
PTP_di _(cmH_2_Oxs)	7.6 (5 to 13.8)	13.4 (10.6 to 19.6)	**0.003**
PTP_di/min_(cmH_2_Oxs/min)	213 (129 to 271)	283 (191 to 369)	**0.026**

C_Ldyn_, dynamic lung compliance; NIV, noninvasive mechanical ventilation; PEEP_dyn_, dynamic positive end-expiratory pressure; PTP_di_, pressure-time product of diaphragmatic pressure per breath; PTP_di/min_, pressure-time product of diaphragmatic pressure per minute; R_L_, pulmonary resistance at mid-inspiratory volume; V_T_, expired tidal volume. The median and interquartile ranges (IQRs) are reported for each variable except respiratory rate which did not deviate from the normal distribution, and for which the mean and standard deviation (SD) are reported. The *P*-values were computed by Wilcoxon's test, except for PS and respiratory rate for which the t-test was applied.

**Figure 1:**
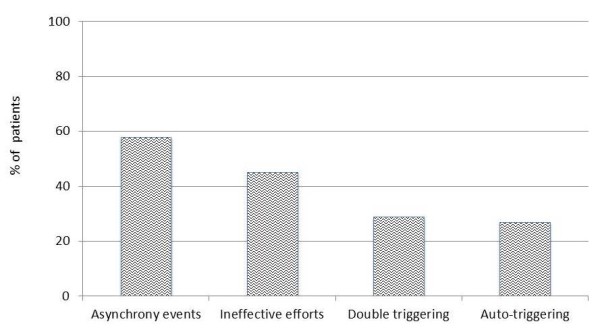
**Distribution of total and single types of asynchrony in overall studied population**.

As shown in Figure 1, any form of asynchrony was present in 58% of the 69 enrolled patients, with IE being by far the most common problem. An AI >10%, indicating severe asynchrony [3], was present in 21/69 (30%) patients, while IE >10% were detected in 14/69 (20%) of patients.

Table 2 presents the occurrence of an AI and IE >10% according to the underlying pathologies. No statistical differences were observed in the distribution of these events. As shown in Table 3, inspiratory effort and respiratory mechanics did not differ between patients with or without an AI >10%; on the other hand, the level of pressure support was significantly higher in the presence of IE >10%. Moreover, tolerance to NIV significantly differ between patients with or without an IE >10%.

**Table 2:** Distribution of the asynchrony index according to the different underlying pathologies

		IE >10%			AI >10%		
		Absent (*n *= 55)	Present (*n *= 14)	*P*	Absent (*n *= 48)	Present (*n *= 21)	*P*
***Pathology***	Restrictive	29 (53)	10 (71)	0.24	24 (50)	15 (71)	0.12
	Obstructive	26 (47)	4 (29)		24 (50)	6 (29)	

AI, asynchrony index; IE, ineffective effort. For each variable counts and column percentages (within brackets) have been reported. *P-*values were computed by Fisher's exact test. For each variable counts and column percentage (within brackets) has been reported. *P-*values were computed by Fisher's exact test.

**Table 3:** Ventilator parameters and respiratory mechanics variables in patients with and without a high prevalence of asynchronies

Variable	IE >10%			AI >10%		
	**Absent** **(*n *= 55)**	**Present** **(*n *= 14)**	***P***	**Absent** **(*n *= 48)**	**Present** **(*n *= 21)**	***P***
						
PS (cmH_2_O)	15 (3)	18 (4.3)	**0.03**	16 (3)	16 (4.4)	0.51
PEEP_e_ (cmH_2_O)	4 (2 to 4)	4 (2 to 5)	0.99	4 (2 to 4)	4 (2 to 5)	0.72
Respiratory rate	21(8)	26 (9)	0.08	21 (7)	24(9)	0.26
V_T_ (ml)	480 (395 to 633)	500 (321 to 620)	0.69	497 (405 to 638)	435 (321 to 610)	0.15
C_Ldyn_ (l/cmH_2_O)	0.061 (0.04 to 0.10)	0.035 (0.03 to 0.07)	0.06	0.06 (0.04 to 0.1)	0.05 (0.03 to 0.11)	0.39
R_L_ (cmH_2_O/l/s)	10 (5.6 to 16.4)	8.3 (5.15 to 16.2)	0.84	10.1 (5.6 to 15.6)	8.6 (5.6 to 19.7)	0.83
PEEP_dyn_ (cmH_2_O)	1.15 (0.6 to 2)	1 (0.8 to 2.9)	0.55	1.2 (0.6 to 2.2)	1.05 (0.6 to 2.8)	0.81
PTP_di_ (cmH_2_Oxs)	12 (7 to 18)	11.8 (6 to 15)	0.46	12 (7.1 to 18.5)	12 (6 to 17)	0.78
PTP_di/min_ (cmH_2_Oxs/min)	257 (152 to 318)	247 (167 to 334)	0.91	258 (150 to 326)	249 (180 to 307)	1
Tolerance	2 (1 to 2)	3 (2 to 3)	**0.006**	2 (1 to 2)	2 (2 to 3)	**0.007**

AI, asynchrony index; C_Ldyn_, dynamic lung compliance; IE, ineffective effort; PEEP_dyn_, dynamic positive end-expiratory pressure; PEEP_e_, external end-expiratory positive pressure; PS, pressure support; PTP_di_, pressure-time product of diaphragmatic pressure per breath; PTP_di/min_, pressure-time product of diaphragmatic pressure per minute; R_L_, pulmonary resistance at mid-inspiratory volume; V_T_, tidal volume measured during spontaneous breathing. The median and interquartile ranges (IQRs) are reported for each variable except PS and respiratory rate which did not deviate from the normal distribution, and for which the mean and standard deviation (SD) are reported. The *P*-values were computed by Wilcoxon's test, except for PS and respiratory rate for which the t-test was applied.

## Discussion

The main findings of this observational study performed in patients with stable respiratory disease ready to be enrolled in a home ventilatory program can be summarized as follows: (i) there is an high prevalence of asynchrony in non-invasively ventilated patients; (ii) the occurrence of asynchrony is not correlated to any variable of respiratory mechanics recorded during spontaneous breathing and does not differ between patients with obstructive or restrictive disease; (iii) the frequency of ineffective efforts is associated with a higher level of pressure support; (iv) an high incidence of asynchronies, above all of ineffective efforts, is associated to a poorer tolerance of NIV.

### Incidence of asynchronies

Several studies have investigated the effect of different ventilator settings on the development of asynchronies, mostly in acutely ill patients. Thille *et al. *[3] showed that approximately one-fourth of invasively ventilated patients had a high incidence of asynchrony during assisted breathing. A high level of pressure support and a large tidal volume were associated with an increased incidence of asynchronies [13]. On the other hand, Vignaux *et al. *[2] recently found an AI >10% in more than 40% of patients during NIV. The level of pressure support and the magnitude of leaks were independent predictive factors of severe asynchronies. The incidence of severe asynchronies was lower in our study (30%) than in the latter studies [2,3], which could be explained by the different ventilators used for NIV. The study by Vignaux *et al. *[2], in fact, was performed in an ICU in which intensive care ventilators were used for NIV with and without the use of an algorithm for compensation for leaks. But it is known, as was recently shown [14], that the asynchrony index is significantly lower with a dedicated NIV ventilator than with ICU ventilators even when the latter are used with their NIV algorithm. In our study, we used a ventilator exclusively designed for NIV.

Concerning the types of asynchronies, we have also confirmed the finding of most of the other studies [2,3,13], that IEs are much more common than either auto-triggering and double triggering.

### Effects of respiratory mechanics

The most important finding of our study was that AI was not different between patients with obstructive or restrictive disease and that none of the parameters of respiratory mechanics was significantly different in those patients showing or not a high frequency of asynchronies. Until now, observational studies have been performed only in a single group of patients (that is, those with COPD) [15] or in a heterogeneous group of patients without subgroup analysis [2,3,13,16]. The relationship between a high level of pressure support and rate of IE was emphasized in COPD patients, due to high lung compliance, which could be responsible for large tidal volumes [13]. It has been shown that in these patients with high compliance, the ventilator continues to inflate the respiratory system long after the inspiratory muscles have ceased to contract and the next inspiratory attempt is likely to occur at a high lung volume, when airway pressure is still markedly positive; the inspiratory effort will not, therefore, always be sufficient to create a pressure gradient capable of being sensed by the ventilator [15,17]. Rather surprisingly, in our study, despite the average lung compliance being higher in COPD patients than in patients with restrictive disease, particularly in those in whom asynchronous events occurred, no correlation was found between lung compliance and the onset of asynchrony. Moreover, we found the same incidence of a high rate of IE both in obstructive and restrictive patients. This could mean that the mechanism rather than the compliance (that is, leaks) could be involved.

PEEP_e _has been shown to decrease ineffective triggering in patients with a high PEEP_dyn _by reducing the portion of Pdi spent to overcome the amount of PEEP_dyn _and needed to trigger the ventilator [18]. On the contrary, other studies [13,16] did not find any influence on the amount of ineffective effort when the PEEP_e _was applied as a "fixed" value (that is, 5 cmH_2_O). In our study, the level of PEEP_dyn_, despite being significantly higher in patients with obstructive respiratory disease, was overall still too small to induce a potential problem of triggering and, therefore, to be the major determinant of the asynchronies described in our patients. Indeed, the fact that overall the patients affected by different underlying diseases showed similar amounts of asynchronies, led us to conclude that there are other potential mechanisms, at least in patients with stable disease. One possible explanation for the relatively high occurrence of asynchrony may be the setting of the ventilator. Nowadays, ever more parameters can be adjusted on the machine, and each of them could be responsible for asynchronies. For example, Haynes *et al. *[19] showed how increasing the rise time while keeping the flow-cycling threshold constant, could significantly reduce tidal volume and, consequently, lower the incidence of IE. The importance of settings was also demonstrated in intubated COPD patients, in whom increasing the expiratory threshold to 70% of the peak inspiratory flow improved patient-ventilator interaction and decreased ineffective efforts without changing inspiratory muscle effort or alveolar ventilation [7].

Since the importance of respiratory mechanics and elevated tidal volume only partially explain the occurrence of asynchronies, we believe that other factors need to be taken into account during NIV. Despite our attention and care to minimize the problem of air leaks, these events may have deeply influenced our results, as already shown [2]. Indeed, even in centers with NIV expertise, it has been shown that the occurrence of leaks is unavoidable [2].

Last, in order to avoid any confounders, such as different settings, we standardized the ventilator adjustments by protocol. Concerning the fixed rise time and expiratory trigger, the latter was automatically set by the ventilator we have used, and has been previously shown to be effective in reducing the effort and improving the ventilatory phase [20]. Moreover, we chose the rise time that in a previous study showed a good balance between the amount of air-leaks and patient tolerance [21]

### Asynchrony and tolerance to NIV

As a secondary outcome, this study showed that those patients with a high incidence of ineffective efforts and asynchronies had also a poorer tolerance of NIV. Until now the association between NIV tolerance and asynchrony was studied only in an acute setting [2].

In this study, performed in patients receiving NIV for acute respiratory failure, the tolerance score was higher in those who showed an AI >10% [2]. However, the impact of a single different type of asynchrony was not studied.

Ineffective effort is a sort of inspiratory muscle effort in a closed system because it is not followed by an activation of the ventilator. This could explain the perception of discomfort with a ventilator when the incidence of this kind of asynchrony is more frequent.

### Clinical implications

As for any physiological study, the main aim of this study was to assess the mechanism(s) of an event, rather than suggesting a clinical practice. The extrapolation of the results obtained in this study for clinical practice is, therefore, questionable, especially because the recordings were performed for a short period during the daytime, while most of the patients requiring home NIV are also ventilated during the night. Having said this, we have shown that the occurrence of asynchronies is very frequent even when the settings are performed in expert centers [2]. A close look at some indirect indices of mismatching (that is, the flow waveform), may help the clinician to detect the most frequent asynchrony between the patient and the machine, that is, ineffective effort [22]. Indeed, it is of clinical interest that, despite the different underlying pathologies, the settings decided by the operators to achieve the same aims (that is, gas exchange amelioration and tidal volume increase) are very similar, and also induced comparable amounts of asynchronies.

### Limitation of the study

As already stated, the gold standard for measuring the interaction between the patient and a ventilator is electromyography of the diaphragm because indirect estimates of the onset and duration of neural inspiratory time based on esophageal pressure and flow could lead to errors, compared to neural inspiratory time measurement. For the same reasons, we were unable to assess the inspiratory trigger delay and the expiratory delay because of the use of the catheter-balloon technique. On the other hand, a good correlation among IE, AT and DT detected from flow/airway pressure and from esophageal pressure signals has been already shown [3,23].

Lastly, we used "only" one type of ventilator and an *a priori *"fixed" setting. However, the ventilator chosen, the Vision, used the same algorithms (above all the "autotrack") present on all the bilevel ventilators produced by Philips-Respironics and widely used for NIV at home (BiPAP Synchrony, BiPAP S/T, BiPAP A30, Trilogy). Moreover, we are aware of the existence of an updated software in the V60. However, a recent bench and clinical study was not able to find any difference between the two generations of ventilator in terms of ineffective trigger, trigger delay and delayed cycling [14]. Of course, the results of the study may not be generalizable and may not represent the "real life" situation in which other different ventilator models are employed, and the clinicians are free to set the ventilatory parameters as they wish.

## Conclusions

Our study shows that in non-invasively ventilated patients with stable respiratory disease, ready to be enrolled in a home care ventilator program, the incidence of asynchrony was relatively high, but does not correlate with any mechanical characteristic of the patient and there is also no difference between patients with obstructive or restrictive disease.

## Key messages

• In stable patients ready to be discharged with a home non-invasive ventilatory program there is a high incidence of asynchrony.

• Thirty percent of patients showed severe asynchrony, most of which were represented by ineffective efforts (detected in 20% of patients).

• The occurrence of asynchrony is not correlated to any variable of respiratory mechanics and does not differ between patients with obstructive or restrictive disease.

• A high incidence of asynchrony is associated with a poorer tolerance of NIV.

## Abbreviations

AI: asynchrony index; AT: auto-triggering; C_Ldyn_: dynamic compliance; COPD: chronic obstructive pulmonary disease; DT: double triggering; ICU: intensive care unit; IE: ineffective effort; IMV: invasive mechanical ventilation; NIV: non-invasive ventilation; P_aw_: airway pressure; P_di_: transdiaphragmatic pressure; PEEP_dyn_: dynamic intrinsic positive end-expiratory pressure; PEEP_e_: positive end-expiratory pressure; P_es_: esophageal pressure; P_ga_: gastric pressure; P_L_: transpulmonary pressure; PS: pressure support; PTP_di_: pressure-time integral of diaphragm; R_L_: pulmonary resistance; T_E_: expiratory time; T_I_: inspiratory time; T_TOT_: total respiratory time; V: flow; V_T_: tidal volume

## Competing interests

All the authors declare that they have no financial and non-financial conflict of interests.

## Authors' contributions

AC carried out the physiological study, collected data and has been involved in drafting the manuscript. LP has made substantial contributions to the acquisition and interpretation of data, and helped to draft the manuscript. PC has made a substantial contribution to conception of the study and the interpretation of data. AM participated in the design of the study and performed the statistical analysis. SN conceived of the study and participated in its design and coordination, and has been involved in revising the manuscript. All authors read and approved the final manuscript.
